# Therapies for COVID-19-Related Persistent Olfactory Disorders: One of the Good Fruits of the Pandemic

**DOI:** 10.3390/pathogens12010072

**Published:** 2023-01-03

**Authors:** Sven Saussez, Luigi Angelo Vaira, Giacomo De Riu, Jérome R. Lechien

**Affiliations:** 1 Department of Human and Experimental Oncology, Faculty of Medicine UMONS Research Institute for Health Sciences and Technology, University of Mons (UMons), B7000 Mons, Belgium; 2 Maxillofacial Surgery Operative Unit, Department of Medicine, Surgery and Pharmacy, University of Sassari, 07100 Sassari, Italy; 3 Biomedical Science Department, PhD School of Biomedical Science, University of Sassari, 07100 Sassari, Italy; 4 Department of Otolaryngology-Head Neck Surgery, Elsan Polyclinic of Poitiers, 86000 Poitiers, France

At the beginning of 2021, the scientific community realized the burden of COVID-19-related persistent olfactory disorders (ODs). The percentage of those infected with COVID-19 who developed severe and persistent ODs [1,2,3] with devastating effects on their quality of life was 5 to 40% [4,5]. 

The impact of this problem was exacerbated by the fact that at the time there were no therapies with proven efficacy for the treatment of post-viral olfactory disorders. The literature lacks randomized trials with appropriate control arms or reliable olfactory assessments. For this reason, all of the systematic reviews published up until now have failed to identify standardized therapeutic protocols [6,7,8,9,10,11]. 

Our article, published in Pathogens in May 2021, was one of the first to investigate the efficacy of the early administration of nasal and systemic corticosteroids in the treatment of COVID-19-related ODs [12]. Corticosteroids are the treatment of choice in ODs related to chronic rhinosinusitis, but their use in ODs related to post-viral etiology was not supported by solid evidence [13,14,15]. The rationale behind the use of corticosteroids in COVID-19-related ODs was based on the preliminary results of a few small-series studies [16], the evidence that patients who had taken corticosteroids to treat their infection had a better recovery [17], and on the hypothesis that the persistence of the OD was related to inflammatory neuropathy at the level of the olfactory epithelium [18,19]. However, the results of our study did not reveal a clear advantage of administering both nasal and systemic corticosteroids during the period of infection. This could have been due to the fact that starting treatment so early meant including patients who would have recovered spontaneously anyway, and therefore some form of selection of cases to be treated was necessary on the basis of risk factors that were never identified with certainty [20,21,22,23].

However, the results of larger randomized trials that included subjects with persistent ODs did not give concordant or negative results [24,25,26,27,28,29]. Rashid et al. [24], in their randomized double-blind placebo-controlled study including 276 COVID-19 patients with anosmia did not find a significant benefit of topically administering betamethasone in terms of the speed of recovery of olfactory function. Kasiri et al. [26] and Hintschich et al. [27] treated patients with persistent ODs after SARS-CoV-2 infection with mometasone furoate as in our study without detecting significant benefits. This was confirmed by the recent meta-analysis of Kim et al. [30] and is likely related to poor delivery of the corticosteroid spray to the olfactory epithelium [31,32]. Similarly, oral corticosteroids also did not demonstrate a clear advantage in terms of olfactory recovery compared to olfactory training alone [28]. For these reasons, the routine prescription of corticosteroids in COVID-19 olfactory loss is currently not recommended [11,33]. Considering the possible side effects, corticosteroids should be prescribed after adequate consideration of the risk/benefit ratio and only in patients with coexisting signs of nasal inflammation [33].

Today, researchers’ efforts are mainly heading in other directions. Olfactory training is the therapy with the most evidence regarding efficacy in improving olfactory function in COVID-19-related persistent ODs [34,35,36]. Given the absence of side effects, olfactory training can be prescribed to all patients with ODs during the period of infection. Other possible therapies described include vitamin A [37], theophylline [37], palmitoylethanolamide and luteolin [38], Omega-3 [39], and platelet-rich plasma [40,41,42].

These great efforts are allowing great strides to be made in studying the efficacy of post-viral OD therapies. Although infectious etiology has always been one of the most frequent causes of persistent olfactory loss, it has often been underestimated or trivialized by both general practitioners and otolaryngologists. This will no longer be possible now that COVID-19 has shed light on this type of disabling dysfunction, raising awareness among patients who will not accept being offered no therapy. This is one of the great lessons that the pandemic has taught us.
